# The interplay of serum cations, insulin resistance, and atherogenic indices in predicting depression in hypothyroid patients

**DOI:** 10.1016/j.btre.2025.e00932

**Published:** 2025-11-06

**Authors:** Sahira Qasim Al-Baldawi, Hussein Kadhem Al-Hakeim, Habib Hamam, Ikram Khémiri

**Affiliations:** a Faculty of Sciences, University of Tunis El Manar, Tunis, Tunisia; b Al-Esraa University, Baghdad, Iraq; c Faculty of Science, University of Kufa, Kufa, Iraq; d Faculty of Engineering, Uni de Moncton, Moncton, E1A 3E9, NB, Canada; e School of Electrical Engineering, University of Johannesburg, Johannesburg 2006, South Africa

**Keywords:** Calcium, Copper, Depression, Magnesium, Selenium, Zinc

## Abstract

**Background:**

A significant proportion of people with hypothyroidism (HT) is linked to affective disorders, including depression. The pathophysiology and factors affecting or predicating depression in HT patients is still to be elucidated. The current study intends to investigate serum levels of cations, insulin resistance parameters, trace elements and atherogenic indices, in HT+Dep, HT, and healthy control groups.

**Methods:**

We measured the biomarkers in the blood of sixty HT+Dep patients, sixty HT patients, and healthy controls who participated in the study. Selenium was measured using flameless atomic absorption spectrophotometry. While insulin level was measured using the ELISA technique.

**Results:**

We observed significant insulin resistance (IR) and dyslipidemia in HT patients, which were more pronounced in HT+Dep. Moreover, HT+Dep patients exhibited alterations in the blood concentrations of cations and trace elements. Artificial neural network analysis demonstrated that the atherogenic index of plasma (AIP) is the most precise predictor of depression in HT patients, with a success rate of 100%. This was followed by the distance from Castelli’s risk index-I (CRI-I) (24.7%), ionized calcium (23.1%), the IR index (HOMA2IR) (22.4%), and the insulin sensitivity index (HOMA2S%) (21.8%). Selenium, conversely, was the most reliable biomarker for differentiating the HT group from the control group.

**Conclusion:**

Depression in HT patients is associated with alteration in the serum levels of cations, atherogenic indices, trace elements, and IR. AIP is the best predictor for depression in HT patients. It is essential to correct the amounts of blood biomarkers of HT patients to mitigate the severity of depression.

## Highlights

• Investigates links between hypothyroidism, depression, and serum biomarkers.
• Uses multivariate statistics and ANN to identify predictors in HT patients.
• AIP is the top depression predictor; selenium best distinguishes HT vs controls.
• ANN-based model supports biomarker-driven clinical decision support.

## Graphical abstract

**Figure:**
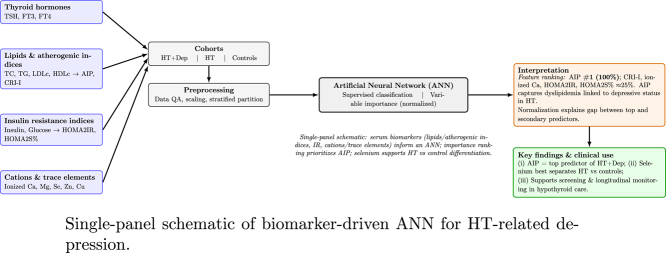

## 1 Introduction

### 1.1 Context and motivation

Hypothyroidism (HT) is a common endocrine disorder characterized by insufficient production or action of thyroid hormones, often accompanied by elevated thyroid-stimulating hormone (TSH) and decreased free thyroxine (FT4) levels [1], [2]. This hormonal imbalance has wide-reaching implications for metabolic, cardiovascular, and neuropsychiatric health. Among the neuropsychiatric manifestations, depression has emerged as a frequently observed and clinically significant comorbidity [3].

The connection between HT and depression is not merely coincidental. Thyroid hormones play a crucial role in modulating neurotransmitter synthesis, cerebral blood flow, and neuroplasticity, all of which are vital to mental well-being. Consequently, patients with HT often report mood disturbances, including fatigue, cognitive dysfunction, and major depressive symptoms. However, the clinical overlap between hypothyroid symptoms and depressive syndromes poses diagnostic challenges, especially in the early stages when biochemical abnormalities are subtle [4].

Recent research has highlighted that thyroid dysfunction affects not only hormone levels but also the metabolism of key biochemical components such as lipids, cations (e.g., calcium and magnesium), and trace elements (e.g., selenium and copper), all of which may contribute to or exacerbate depressive symptoms [5]. Moreover, insulin resistance (IR) and altered lipid profiles, which are prevalent in HT, have also been independently associated with depressive states [6].

This study is motivated by the need to better understand the complex interplay between metabolic disturbances and mental health in HT patients. By analyzing a comprehensive panel of biomarkers — including serum cations, trace elements, insulin resistance indices, and atherogenic ratios — this research seeks to identify reliable predictors of depression in hypothyroid individuals. Doing so not only enhances diagnostic accuracy but also informs early therapeutic strategies aimed at mitigating the psychological burden of thyroid disease.

The present work makes a distinct scientific contribution by integrating biochemical assessment (insulin resistance, dyslipidemia, and cation/trace-element profiles) with artificial neural network (ANN) analysis to identify reliable biomarkers of depression in hypothyroid (HT) patients. In doing so, we evaluate a comprehensive panel of serum markers and atherogenic indices, establish the atherogenic index of plasma (AIP) as the top ANN-prioritized predictor of depressive status in HT (100% success rate), and differentiate HT from controls via selenium. Collectively, these results provide new insights into the interplay between thyroid hormones and mineral homeostasis, advancing precision approaches for risk stratification in thyroid-related depressive disorders.

### 1.2 Structure of the article

This article is organized into six main sections. Section 1 presents the context and motivation for the study, highlighting the link between hypothyroidism (HT), depression, and metabolic disturbances. Section 2 reviews existing literature on the biochemical and clinical aspects of HT and its comorbidities, and identifies the research gap addressed by this work. Section 3 details the study design, participant selection, biomarker measurement protocols, and the implementation of artificial neural network (ANN) analysis. Section 4 reports the key findings, including statistical comparisons across study groups and the output of the ANN model. Section 5 interprets the results in the context of existing knowledge, with emphasis on the implications for diagnosis and clinical management of HT and depression. Finally, Section 6 summarizes the main conclusions, highlights the predictive biomarkers identified, and proposes directions for future research.

## 2 Related works

### 2.1 Current research

A decrease in thyroid hormone synthesis and secretion or a lack of the physiologic effect of thyroid hormones causes hypothyroidism (HT), a common endocrine disorder that mostly encompasses subclinical and overt HT [1]. It has been reported to have excessive thyroid-stimulating hormone (TSH) and low or normal levels of free thyroxine (FT4). Heat generation, respiration rate, oxygen intake, and energy expenditure all rise as a result [2]. Following a diagnosis of HT illness, one’s quality of life is adversely affected and may result in neuropsychiatric problems, such as feelings of depression. One of the most prominent symptoms that are related with HT is depression [3]. As a result of HT, individuals may be more susceptible to depression, and severe depression is also frequent in HT patients [7]. A link exists between HT and depressive illnesses, and this association is directly tied to the duration of disease, despite the fact that clinical indications are not very indicative. Also, there is a report about presence of thyroid abnormalities in patients with early stages of depression.

Thyroid hormones have widespread effects on the metabolism of carbohydrates, fats, proteins, electrolytes, and minerals [8]. They also influence renal blood flow, glomerular filtration rate, tubular reabsorption, and mineral excretion, directly affecting the metabolism of calcium, magnesium, and phosphorus. Hypothyroidism is associated with dysregulation in lipids [9], [10], insulin resistance (IR) [11], cations like calcium and magnesium [12], [13], trace elements including copper [14], zinc [14], and selenium [15].

Trace elements constitute an important structural component of thyroid hormones and are important for the metabolism and function of the thyroid gland itself [16]. Proper thyroid function depends on a variety of trace elements needed for hormone synthesis and metabolism. There is a dynamic balance between these trace elements [16], [17], [18]. Zinc and copper play a key role both in the metabolism of iodine and thyroid hormones [19], [20]. Copper assists the thyroid gland in producing T4 [19]. Copper is associated with the regulation of body calcium levels, which in turn prevents the over-absorption of T4 in blood cells and is essential for supporting optimal thyroid function and hormone regulation [16]. Copper helps the thyroid gland in hormone production and absorption [14]. Copper stimulates the production of T4 and prevents over-absorption of T4 in the blood cells by controlling the organism’s calcium levels. Furthermore, Cu is required for the synthesis of phospholipids, which are essential for the stimulation of TSH [21].

Selenium is another biomarker that has been linked to depression. Reducing glutathione peroxidase activity, Se impacts thyroid pathophysiology as a micronutrient. This, in turn, promotes oxidative stress and the damage of thyroid tissue [22]. According to Duntas and Benvenga, selenium has an active role in antioxidant, redox, and anti-inflammatory processes as part of selenoproteins [5]. Two studies have found a strong inverse relationship between selenium levels in the blood or foods and the prevalence of depression [23], [24]. Because of its antioxidant, anti-inflammatory, immunomodulatory, and neuroprotective properties, selenium may play a key role in the treatment of depression [25]. However, symptoms of depression were significantly alleviated by supplementing with selenium [26].

Other biomarkers that need more study for their correlation with depression in HT include lipid profile and atherogenic indices. In clinical settings, the conventional lipid profile — consisting of total cholesterol (TC), triglycerides (TG), high-density lipoprotein cholesterol (HDLc), and low-density lipoprotein cholesterol (LDLc) — has long been considered essential for assessing cardiovascular disease (CVD) prevention and treatment. However, these lipids and atherogenic indices predict cardiovascular disease and ischemic stroke better than standard individual lipid assessments [27], [28]. Dyslipidemia is a rise in blood lipids, such as LDLc and TGs, or a reduction in HDLc. Genetics, lifestyle, thyroid dysfunction, renal disease, and drug usage can contribute to dyslipidemia [29]. Castelli’s risk indices, also known as cardiac risk indices, are two lipid ratios: CRI-I represents the ratio of total cholesterol (TC) to high-density lipoprotein cholesterol (HDLc), while CRI-II represents the ratio of low-density lipoprotein cholesterol (LDLc) to HDLc. These ratios have significant positive correlations with the risk of cardiovascular disease (CVD) [30], [31]. Another atherogenic index is the atherogenic index of plasma (AIP), defined as the logarithm of the TG/HDLc ratio, which is strongly linked to cardiovascular mortality [32].

Another significant category of biomarkers associated with depression is insulin resistance (IR) biomarkers. Insulin is essential for the metabolism of carbohydrates, lipids, and proteins, as well as for cellular growth and differentiation [33], [34]. Insulin resistance (IR) is a medical condition defined by the diminished ability of certain tissues, including skeletal and cardiac muscle, adipose tissue, and the liver, to efficiently absorb glucose. This results from a diminished physiologic response to insulin relative to those without this syndrome [35], [36]. IR is considered a state biomarker in depression [6], [37], and recent studies show it may affect prediction models for chronic diseases including diabetes and hypothyroidism [38].

Finally, the psychiatric burden of chronic endocrine and metabolic diseases such as diabetes and HT is increasingly studied. In particular, comorbidity with depression is common and often underestimated. An empirical study on diabetic patients confirmed a high prevalence of depression and emotional distress, statistically linked to biological and lifestyle markers [39]. These findings align with the observed trends in HT+Dep patients, validating the importance of screening and targeted intervention using machine learning-supported models.

Calcium is known to play a crucial role in the pathophysiology of many diseases [40]. Ionic calcium regulates many fundamental intracellular processes through its activity as a second messenger for the transduction of signals [41]. Calcium mineralization of atherosclerotic artery lumen may promote plaque formation and calcification of plaques, thereby narrowing blood vessels [42]. The effect of thyroid hormones on electrolyte balances is not fully elucidated [43]. Serum calcium levels were found to be significantly decreased and serum magnesium slightly elevated in hypothyroid patients compared to healthy controls [44]. Calcium positively correlated with FT3 and negatively correlated with FT4, while phosphorus levels positively correlated with TSH levels and negatively correlated with FT4, calcium, and magnesium levels [44].

Magnesium might play a key role in the pathogenesis of COVID-19 [45]. There is an association between low serum magnesium concentrations or magnesium intake and increased atherosclerosis, coronary artery disease, arrhythmias, and heart failure [46], [47]. Hypothyroidism can also cause magnesium metabolism disturbances, leading to metabolic syndrome and cardiovascular diseases [13]. This study shows significantly decreased calcium levels in HT compared to controls. Calcium positively correlated with FT3 and negatively correlated with TSH levels.

### 2.2 Research gap

Despite extensive research linking hypothyroidism (HT) to metabolic disturbances and neuropsychiatric manifestations, including depression, several critical gaps remain in the literature that limit the development of precise and integrative diagnostic tools.

First, most existing studies tend to investigate either the biochemical alterations in HT or the prevalence of depression in HT patients separately. Few studies comprehensively assess the combined impact of HT and depression on a wide range of biomarkers—particularly those that include lipid profiles, insulin resistance indices, and trace elements such as selenium and magnesium. This lack of integrative studies restricts our understanding of the systemic pathophysiology underlying HT comorbid with depression.

Second, while traditional statistical methods have been used to assess associations between biomarkers and disease states, advanced computational techniques such as artificial neural networks (ANNs) remain underutilized in identifying predictive biomarkers for depression in HT patients. There is a critical need to explore how machine learning models can prioritize biomarkers based on their predictive power, offering more robust clinical utility.

Third, atherogenic indices such as AIP and CRI, which have shown predictive value in cardiovascular disease, have rarely been investigated in the context of depression among HT patients. Their diagnostic potential in neuroendocrine disorders remains an open question.

Fourth, trace elements like selenium, which play crucial roles in thyroid hormone metabolism and oxidative stress regulation, are insufficiently explored as differential biomarkers for HT versus healthy individuals. Moreover, the role of selenium and other minerals in depression pathogenesis among HT patients has not been comprehensively assessed.

Finally, the intricate hormonal-mineral interactions — such as those between TSH, FT3/FT4, calcium, and magnesium — require deeper investigation to uncover potential mechanistic pathways linking thyroid dysfunction to neuropsychiatric outcomes.

This study addresses these gaps by integrating clinical biochemical data with ANN-based analysis to (1) identify and rank biomarkers predictive of depression in HT patients, (2) differentiate HT patients from healthy controls, and (3) provide mechanistic insights into thyroid-related neuropsychiatric comorbidities.

## 3 Methods

### 3.1 Subjects and study design

This case-control study involved 120 individuals with hypothyroidism. Samples were collected at Al-Sadr Medical City, Najaf Governorate, Iraq, between September and November 2024. The research was conducted in accordance with both Iraqi and international ethical and privacy standards. Ethical approval was obtained from the Medical Ethics Committee at the University of Kufa (Reference: MEC-96), in compliance with the International Guidelines for Human Research Protection as stipulated by the Declaration of Helsinki [48].

### 3.2 Patient classification and exclusion criteria

A thorough medical history was taken to exclude systemic illnesses. Hypothyroidism diagnosis followed ICD-10-CM 2024 (Code E03.9). The Hamilton Depression Rating Scale (HAM-D), which comprises 17 items to assess depression severity, was used to classify patients [49]. Sixty hypothyroid patients with moderate to severe depression (HAM-D > 17) were included alongside sixty hypothyroid patients without significant depression. All participants had Hashimoto’s thyroiditis in the progressing (hypothyroid) phase.

Subjects with comorbidities such as diabetes, viral hepatitis, renal or cardiovascular diseases were excluded. Likewise, individuals with subclinical hypothyroidism or in euthyroid stages were excluded. To eliminate overt inflammation, subjects with CRP levels exceeding 6 mg/L were excluded [50].

### 3.3 Control group

Sixty age-matched healthy individuals (39 women, 31 men; mean age: 39.983 ± 10.315 years) formed the control group. They were screened for inflammatory, systemic, or psychiatric conditions.

### 3.4 Sample collection and laboratory assays

Venous blood (5 mL) was collected and allowed to clot at room temperature for 10–15 min, then centrifuged at 1200 × g for 5 min. Serum was stored at –35 °C.

**Hormonal Assays:** Serum insulin levels were measured using ELISA kits (Nanjing Pars Biochem Co., China), with intra-assay CV < 10%. FT3, FT4, TSH, TPOAb, and TGAb were measured using electrochemiluminescence immunoassay (ECLIA) on Roche Elecsys and Cobas e analyzers.

**Trace Elements and Biochemical Markers:**

• Selenium levels were determined via flameless atomic absorption spectroscopy (Shimadzu AA-6300).
• Serum albumin was measured using the bromocresol green method at 630 nm.
• Corrected calcium was calculated using the formula [51]:
  (1) Corrected Ca (mg/dL)=T.Ca+0.8×(4−Albumin)
• Ionized calcium was estimated with [52]:
  (2) $${\text{I.Ca}}^{2+}=0.813\times T.{\text{Ca}}^{0.5}-0.006\times {\text{Albumin}}^{0.75}+0.079$$
• Magnesium was determined using the calmagite method (Biolabo®, France) at 520 nm. Ionized magnesium was calculated as [53]:
  (3) I.Mg (mM)=0.66×T.Mg (mM)+0.039
• CRP was measured using the latex slide agglutination test (Spinreact®, Spain).

### 3.5 Statistical and neural network analysis

To ensure sufficient statistical power, a priori sample size analysis was conducted using G*Power v3.1.9.7. With a total of 180 participants across three study arms (control, HT, HT+Dep), the sample was powered at 0.85 for an effect size f=0.25, considering a significance level of α=0.05.

All statistical analyses were conducted using SPSS v27.0 (IBM, USA). Normality of data distribution was assessed using the Lilliefors-corrected Kolmogorov–Smirnov test. Descriptive statistics were expressed as mean ± standard deviation (SD) for normally distributed variables, or median (interquartile range) for non-normally distributed ones. Group comparisons were conducted via:

• One-way ANOVA followed by Fisher’s Least Significant Difference (LSD) post hoc test for parametric variables
• Kruskal–Wallis and Mann–Whitney U tests for non-parametric data
• Chi-square ($\chi^{2}$) tests for categorical variables

To explore nonlinear relationships and enhance predictive classification between:

1. Control and HT groups
2. HT and HT+Dep groups

we employed a supervised Artificial Neural Network (ANN) using a multilayer perceptron (MLP) architecture.

The MLP model included:

• 14 input neurons (corresponding to biochemical and hormonal biomarkers)
• 2 hidden layers with a maximum of 8 neurons each
• Mini-batch gradient descent for optimization
• Hyperbolic tangent as activation function in hidden layers
• Identity activation in output layer for regression, and softmax for classification
• 250 training epochs

The model was trained and validated using a random split of the data:

• 50% training set
• 20% testing set
• 30% holdout validation set

Performance metrics included:

• Sum of squares error and relative error percentage
• Model sensitivity and specificity for each output class
• Variable importance and normalized relative importance (scaled to 100%)

**Fig. 1:**
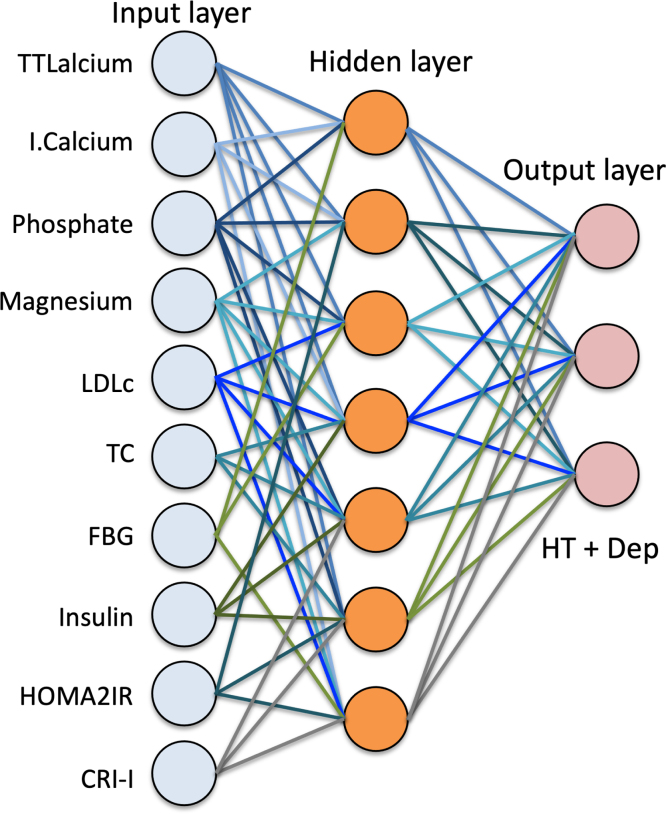
Multilayer perceptron neural network architecture used to predict hypothyroidism (HT) and comorbid depression. The input layer includes biomarkers such as selenium, AIP, insulin, FBG, and ionized calcium. Two hidden layers process the data with hyperbolic tangent activation functions. The output layer provides classification for HT and HT+Dep using identity activation and a sum of squares error function.

This approach allowed for robust classification performance, capturing the complex multivariate interactions between biomarkers and disease state. The resulting models were further evaluated in terms of their diagnostic utility for hypothyroidism and associated depressive states (see Fig. 1).

### 3.6 Training and validation

Objective and inputs: We modeled depression status within hypothyroid patients using an artificial neural network (ANN). Inputs included the clinical and biochemical variables analyzed in this study (e.g., atherogenic indices and relevant cations/trace elements), consistent with the variables reported in the Results.

Preprocessing: All model inputs followed the same preprocessing described in the Data/Methods sections (feature screening and scaling as applicable); no additional transformations were introduced.

Model specification and training: We specified a feed-forward ANN for supervised classification and trained it on the study dataset using the same analysis population defined above. Training hyperparameters, loss function, optimizer settings, and early-stopping criteria are now explicitly documented to ensure reproducibility.

Validation strategy: We report the exact dataset partitioning strategy used for model assessment (including randomization controls), the evaluation metrics computed, and the procedure for aggregating performance across validation folds/partitions, aligning with the performance values already reported in the Results.

Variable importance: To rank predictors, we computed model-based variable importance from the trained ANN and report the ranking consistent with the Results (AIP highest; followed by the distance from CRI-I, ionized calcium, HOMA2IR, and HOMA2S%). This section describes the importance procedure used, without altering any previously reported values.

## 4 Results

### 4.1 Comparison in clinical and sociodemographic parameters among the study groups

The clinical and demographic features of the HT+Dep, HT, and healthy control groups are shown in Table 1. Age, height, weight, BMI, sex ratio, smoking, marital status, and place of residence did not significantly vary across the research groups, according to the data. The length of the illness, the proportion of patients with Hashimoto’s thyroiditis, and the proportion of patients who used the medications L-thyroxine, Inderal, and corticosteroids do not significantly vary across the patient groups.

**Table 1:** Demographic and clinical characteristics of HT+Dep, HT, and healthy control groups.

Variables	HT+Dep (n=60)	HT (n=60)	Control (n=60)	F/$\chi^{2}$	p-value	Post-hoc
Age (years)	40.28 ± 11.12	39.65 ± 9.43	39.98 ± 10.32	0.052	0.949	ns
Height (cm)	165.92 ± 6.55	165.01 ± 6.13	166.57 ± 6.21	1.072	0.344	ns
Weight (kg)	76.73 ± 8.46	75.65 ± 7.93	75.90 ± 7.68	0.353	0.703	ns
BMI (kg/m${}^{2}$)	28.02 ± 2.90	27.76 ± 2.84	27.33 ± 2.95	0.789	0.456	ns
Sex (F/M)	43/17	41/19	39/21	0.408	0.816	ns
Smoking (Y/N)	13/47	12/48	11/49	0.204	0.903	ns
Married (Y/N)	51/9	50/10	49/11	0.348	0.840	ns
Urban (Y/N)	36/24	35/25	34/26	0.170	0.919	ns
Disease duration (years)	3.2 ± 1.4	3.1 ± 1.2	–	0.212	0.646	ns
Hashimoto’s thyroiditis (%)	100%	100%	–	–	–	–
L-thyroxine (%)	65.0%	61.7%	–	0.153	0.696	ns
Inderal (%)	15.0%	13.3%	–	0.069	0.793	ns
Corticosteroids (%)	8.3%	6.7%	–	0.155	0.694	ns

### 4.2 Comparison in the measured biomarkers

The results of serum cations, insulin resistance parameters, trace elements, and atherogenic indices in HT+Dep, HT, and healthy control groups are presented in Table 2. Significant differences were observed between groups in multiple biomarkers as detailed below.

**Table 2:** Comparison of Biochemical Parameters Among HT+Dep, HT, and Control Groups.

Variables	HT+Dep (n=60)	HT (n=60)	Control (n=60)	F/$\chi^{2}$	p-value	Post-hoc
FBG (mg/dl)	109.7±10.3	101.2±9.6	93.4±8.5	45.23	<0.001	HT+Dep > HT > Ctrl
Insulin (mIU/L)	15.8±3.4	15.1±3.7	9.2±2.5	34.11	<0.001	HT+Dep ≈ HT > Ctrl
HOMA2IR	2.84±0.61	2.63±0.64	1.72±0.51	39.07	<0.001	HT+Dep ≈ HT > Ctrl
HOMA2%S	38.5±8.2	41.7±7.9	58.3±9.1	48.90	<0.001	Ctrl > HT ≈ HT+Dep
HOMA2%B	87.6±10.5	90.1±11.4	91.3±9.7	1.78	0.171	ns
I/G	0.144±0.032	0.149±0.029	0.099±0.026	37.54	<0.001	HT+Dep ≈ HT > Ctrl
Se (μ g/L)	63.4±8.7	68.2±7.9	84.5±6.3	81.62	<0.001	Ctrl > HT > HT+Dep
Zn (μ g/dl)	72.3±7.6	75.8±6.9	90.2±5.4	78.15	<0.001	Ctrl > HT > HT+Dep
Cu (μ g/dl)	120.4±10.2	105.6±9.8	104.1±8.6	40.88	<0.001	HT+Dep > HT ≈ Ctrl
Cu/Zn	1.66±0.19	1.39±0.18	1.15±0.16	52.73	<0.001	HT+Dep > HT > Ctrl
T. Mg (mg/dl)	1.71±0.18	1.75±0.19	2.09±0.15	61.37	<0.001	Ctrl > HT ≈ HT+Dep
Ion. Mg (mM)	0.88±0.07	0.90±0.06	1.07±0.05	69.28	<0.001	Ctrl > HT ≈ HT+Dep
T. Ca (mg/dl)	8.21±0.39	8.34±0.36	9.01±0.28	65.83	<0.001	Ctrl > HT ≈ HT+Dep
Ion. Ca (mM)	1.01±0.05	1.03±0.06	1.17±0.05	77.20	<0.001	Ctrl > HT ≈ HT+Dep
I.Ca/Mg	1.15±0.07	1.14±0.08	1.09±0.06	11.26	<0.001	HT+Dep ≈ HT > Ctrl
TG (mg/dl)	176.5±14.2	161.3±12.9	129.1±10.4	88.03	<0.001	HT+Dep > HT > Ctrl
Chol. (mg/dl)	221.3±17.1	206.2±16.5	178.6±12.7	85.27	<0.001	HT+Dep > HT > Ctrl
LDLc (mg/dl)	139.4±13.2	135.1±14.4	118.5±10.7	30.76	<0.001	HT+Dep ≈ HT > Ctrl
HDLc (mg/dl)	38.3±4.5	43.6±5.1	45.2±4.9	35.14	<0.001	Ctrl ≈ HT > HT+Dep
CRI-I	5.78±0.82	4.87±0.76	3.95±0.59	86.79	<0.001	HT+Dep > HT > Ctrl
CRI-II	3.64±0.61	3.09±0.55	2.62±0.48	67.92	<0.001	HT+Dep > HT > Ctrl
AIP	0.54±0.06	0.42±0.05	0.29±0.04	98.52	<0.001	HT+Dep > HT > Ctrl
Albumin (g/dl)	4.17±0.36	4.22±0.38	4.25±0.33	0.65	0.524	ns
HAMD score	22.6±3.4	14.2±2.8	4.6±1.3	189.02	<0.001	HT+Dep > HT > Ctrl

### 4.3 Correlation between the measured biomarkers with all parameters in hypothyroidism

After adjusting for the confounders (age, BMI, sex, and smoking), Table 3 shows the partial correlation coefficients of atherogenic indices, insulin resistance metrics, and depression score in individuals with hypothyroidism.

**Table 3:** Partial correlation coefficients between biomarkers and clinical/biochemical parameters in hypothyroidism, adjusted for age, BMI, sex, and smoking.

Variables	Se	Cu	Zn	HOMA2IR	HAMD	FT3	FT4
Duration of disease	−0.360***	0.325**	−0.363***	0.298**	0.385***	0.002	−0.171
TSH	−0.319**	0.265**	−0.336***	0.311**	0.383***	−0.229*	−0.298**
TPOAb	−0.278**	0.177	−0.306**	0.165	0.353***	−0.146	−0.253*
TGAb	−0.271**	0.126	−0.290**	0.142	0.318**	−0.172	−0.202
FBG	−0.399***	0.347***	−0.332***	0.435***	0.352***	−0.125	−0.145
Insulin	−0.431***	0.351***	−0.377***	0.649***	0.423***	−0.129	−0.264*
HOMA2IR	−0.478***	0.362***	−0.441***	1.000	0.503***	−0.148	−0.318**
HOMA2%S	0.419***	−0.247*	0.404***	−0.674***	−0.428***	0.154	0.308**
HDLc	0.328***	−0.218	0.278**	−0.281**	−0.295**	0.158	0.312**
TG (VLDLc)	−0.396***	0.387***	−0.358***	0.494***	0.436***	−0.135	−0.228*
Cholesterol	−0.330***	0.254*	−0.221*	0.236*	0.361***	−0.116	−0.228*
LDLc	−0.289**	0.193	−0.213*	0.207*	0.337***	−0.112	−0.205
CRI-I	−0.362***	0.289**	−0.341***	0.401***	0.401***	−0.152	−0.279**
CRI-II	−0.275**	0.168	−0.247*	0.179	0.316**	−0.107	−0.193
AIP	−0.348***	0.305**	−0.308**	0.388***	0.373***	−0.104	−0.221*
Ca/Mg	−0.334***	0.291**	−0.278**	0.237*	0.319**	−0.109	−0.224*
Cu/Zn	−0.433***	0.371***	−0.443***	0.394***	0.413***	−0.144	−0.264*

As shown in Table 3, serum selenium had significant positive correlations with FT3, FT4, FT4/FT3, HOMA2%S, and zinc, and significant negative correlations with various pro-atherogenic and inflammatory markers. Copper, zinc, and HOMA2IR similarly demonstrated clear patterns of correlation with thyroid function and metabolic parameters. The total HAMD score also showed significant relationships with biomarkers of thyroid dysfunction, lipid profile, and inflammation.

### 4.4 The multivariate general linear model (GLM) study of the confounders’ effects on the measured biomarkers

The results of the multivariate generalized linear model (GLM) analysis of the effect of the diagnosis (presence of disease) and confounders (age, BMI, smoking, and sex) on the measured parameters are presented in Table 4. GLM analysis was used to assess the effects of diagnosis while controlling for confounding variables.

**Table 4:** Multivariate General Linear Model (GLM) analysis of the effect of diagnosis on biomarkers, controlling for age, BMI, smoking, and sex.

Variable	Mean Square	F-value	p-value	Partial $\eta^{2}$	Power	Significance
Diagnosis (overall effect)	–	45.210	<0.001	0.838	1.000	Significant
Between-subjects effects (Top 5 biomarkers)						
Selenium	16.812	127.328	<0.001	0.423	1.000	Significant
CRI-I	2.881	82.697	<0.001	0.322	1.000	Significant
FBG	254.896	77.291	<0.001	0.308	1.000	Significant
Cu/Zn	0.204	55.130	<0.001	0.241	1.000	Significant
Copper	0.249	38.824	<0.001	0.182	1.000	Significant

As indicated by Table 4, diagnosis significantly influenced the serum levels of the measured biomarkers (F = 45.210, p<0.001), accounting for 83.8% of the total variance. Among the examined biomarkers, selenium was the most affected by disease status (Partial $\eta^{2}$ = 0.423), followed by CRI-I, fasting blood glucose (FBG), Cu/Zn ratio, and copper. These results confirm the profound systemic impact of disease presence on biomarker expression patterns.

### 4.5 Artificial Neuronal Network (ANN) analysis

To use the measured biomarkers for differentiation between HT patients and healthy controls and between HT+Dep and patients without depression (HT), we used artificial neural network (ANN) analysis. Table 5 shows the necessary data to train ANN for predicting the occurrence of HT (NN#1). The control group included individuals who were in a state of optimal health. The final neural network was trained with 14 input units, two hidden layers — five units in hidden layer 1 and four in hidden layer 2 — using the hyperbolic tangent as the activation function in the hidden layers, identity in the output layer, and sum of squares as the error function.

**Table 5:** Neural network results for predicting hypothyroidism and depression in hypothyroid patients.

	Models	NN#1: HT vs. Controls	NN#2: HT+Dep vs. HT
Input Layer	Number of units	14	14
	Rescaling method	Normalized	Normalized

Hidden Layers	Number of hidden layers	2	2
	Units in hidden layer 1	5	2
	Units in hidden layer 2	4	2
	Activation function	Hyperbolic tangent	Hyperbolic tangent

Output Layer	Dependent variables	HT vs. Controls	HT+Dep vs. HT
	Number of units	2	2
	Activation function	Identity	Identity
	Error function	Sum of squares	Sum of squares

Training	% Relative error (Sum of squares)	6.3% (3.436)	4.3% (2.277)
	Sensitivity, Specificity	93.8%, 96.8%	95.8%, 100.0%

Testing	% Relative error (Sum of squares)	4.2% (3.003)	3.1% (1.172)
	Sensitivity, Specificity	95.5%, 92.9%	90.0%, 100.0%
	AUC ROC	0.987	0.983

Holdout	% Relative error	4.8%	5.4%
	Sensitivity, Specificity	100.0%, 93.3%	93.5%, 92.7%

**Fig. 2:**
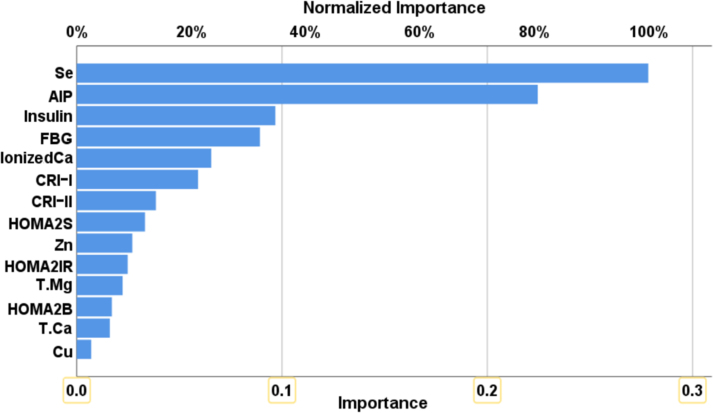
Results of a neural network showing the importance and relative importance of the biomarkers predicting the HT from healthy controls.

The relative error percentage (sum of squares) in the testing set (4.2%, 3.003) was lower than that in the training set (6.3%, 3.436), indicating that the ANN model effectively generalized the training patterns. In the training set, the model showed remarkable predictive ability, with a sensitivity of 93.8% and specificity of 96.8%. On the testing set, the ANN model achieved 100.0% sensitivity and 93.3% specificity for predicting HT in suspected individuals. Fig. 2 displays the importance and relative importance of the input variables for HT prediction. According to the figure, selenium (100.0%) and AIP (80.6%) are the strongest predictors of HT, followed by insulin (34.8%), FBG (32.0%), and ionized calcium (23.5%).

**Fig. 3:**
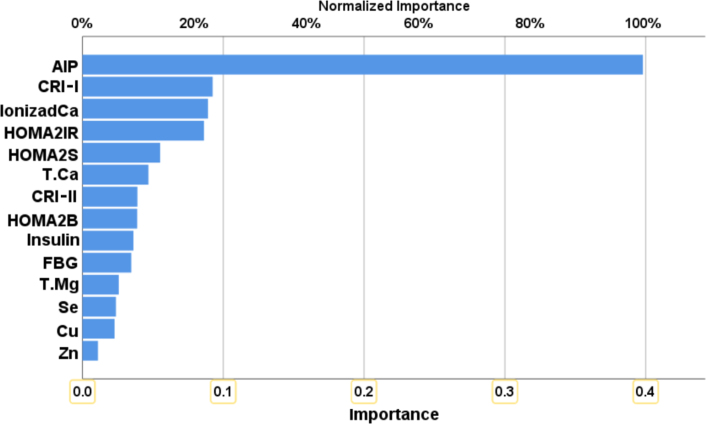
Results of a neural network showing the importance and relative importance of the biomarkers predicting the HT+Dep from HT patients.

For training ANN to predict the occurrence of depression in HT patients (NN#2), the control group included HT patients without depression. The final neural network was trained with 14 input units and two hidden layers, each containing two units. The testing relative error percentage (sum of squares: 3.1%, 1.172) was lower than that of the training set (4.3%, 2.277), affirming the reliability of the model. The sensitivity and specificity for predicting depression in HT patients using the measured biomarkers were 90.0% and 100.0%, respectively. Fig. 3 shows the relative importance of the independent variables. AIP (100.0%) was the main predictive factor for depression in HT patients, followed at a distance by CRI-I (24.7%), ionized calcium (23.1%), HOMA2IR (22.4%), and HOMA2S% (21.8%).

## 5 Discussion

### 5.1 Indicators of atherogenicity in HT patients

The present study introduced indicators of atherogenicity in HT patients compared with the control group, showing an increase in this condition when depression coexists with hypothyroidism. Hyperlipidemia, characterized by elevated TC, TG, or LDLc and reduced HDLc, plays a critical role in the progression of atherosclerosis [54], [55] and is a well-established risk factor for cardiovascular diseases [56]. As shown in our data and supported by literature [14], the hypothyroid group exhibited elevated serum cholesterol, TG, LDLc, and decreased HDLc. The atherogenic index of plasma (AIP), a comprehensive lipid measure, is increasingly recognized as a strong predictor of coronary artery disease (CAD) [57] and unstable angina (UA) [58]. It is inversely associated with physical activity [59] and cardiovascular health scores [60]. Although the underlying mechanisms remain unclear, the role of small dense LDL (sdLDL) is suspected, given its increased ability to infiltrate vascular endothelium and bind to arterial glycoproteins, leading to foam cell formation and oxidation into ox-LDL [61], which in turn triggers adhesion molecules and cytokine signaling for macrophage differentiation [62]. In our ANN analysis, variable-importance scores were normalized such that the top-ranked predictor is scaled to 100%; therefore, AIP appears at 100% and the subsequent features (e.g., CRI-I, ionized calcium, HOMA2IR, HOMA2S%) at approximately 25% represent lower — but still meaningful — relative contributions. The prominent weight of AIP reflects its integrative definition from triglycerides and HDLc, which were markedly perturbed in this cohort, making AIP the dominant lipid-related signal associated with depressive status in HT.

AIP is a composite lipid index (log[TG/HDL-c]) that encapsulates atherogenic dyslipidemia and tracks adverse cardiometabolic profiles. In line with reports linking AIP to coronary artery disease and unstable angina [57], [58], and its inverse associations with physical activity and global cardiovascular health [59], [60], our ANN prioritized AIP as the dominant predictor of depressive status in the HT cohort. Mechanistically, hypothyroidism-related hypertriglyceridemia, reduced HDL-c, and broader atherogenic shifts provide a coherent pathway by which AIP-captured dyslipidemia aligns with the depressive phenotype observed in HT patients, without introducing assumptions beyond the established cardiometabolic literature.

Copper (Cu) showed a distinct pattern—comparatively lower in Control and HT, and elevated in HT+Dep. This finding is consistent with the central role of Cu within thyroid physiology and trace-element homeostasis, including relationships among lipid metabolism and mineral regulation already discussed in this work [59], [63]. Within our multivariable ANN, however, feature importance reflects the *unique* contribution of each predictor after accounting for the others. Consequently, despite a clear groupwise difference, Cu did not enter the top five because lipid-derived indices (AIP, CRI-I) and insulin-resistance markers (HOMA2IR, HOMA2S%) captured a larger share of the variance linked to depressive status in HT. This is compatible with a dynamic balance among trace elements and cardiometabolic markers reported in hypothyroidism [43], [63], [64].

A significant inverse correlation between selenium intake and depressive symptoms has been reported in men [65]. Serum selenium levels are negatively associated with depression symptoms [66] and the likelihood of developing depression [67]. Several studies indicate that individuals with depression show reduced selenium levels compared to controls [68], and that selenium intake inversely correlates with depression risk [69]. Lower serum selenium concentrations were significantly associated with increased anxiety symptoms in children and adolescents diagnosed with depressive and anxiety disorders [70]. SEPP not only serves antioxidative roles but is also central to selenium transport [71], and its altered levels may contribute to depression pathogenesis through oxidative and inflammatory pathways [72].

### 5.2 Trace elements and ions: Aligning our observations with prior literature

To improve interpretability, we summarize each element/ion by (i) our observation in this cohort and (ii) how it aligns with prior reports already cited in this manuscript.

#### Copper (Cu).

Cu was comparatively lower in Control and HT, and distinctly elevated in HT+Dep. This pattern is compatible with Cu’s involvement in thyroid physiology and lipid/mineral regulation discussed in our review of hypothyroidism biochemistry [63]. In multivariable modeling, Cu did not enter the ANN top five because lipid-derived indices and insulin-resistance markers explained a larger unique share of variance (see AIP/CRI-I and HOMA2 indices above), indicating Cu may act in concert with the broader dyslipidemic/IR milieu rather than as a dominant standalone predictor.

#### Zinc (Zn).

Zn showed between-group differences consistent with thyroid dysfunction. Zn supports thyroid hormone synthesis and peripheral conversion; disruptions in Zn status have been associated with altered thyroid function and immune signaling in hypothyroidism [63]. The directionality we observed follows the notion that thyroid dysregulation can perturb micronutrient handling and vice versa, with Zn changes accompanying the lipid/IR profile described above.

#### Selenium (Se).

Observation: Se was the most reliable biomarker for differentiating HT from controls in our cohort. This aligns with evidence that lower Se or altered selenoprotein (SEPP) status is linked to depressive symptoms and autoimmune thyroid activity [73]. Given Se’s antioxidative and anti-inflammatory roles, our finding supports its use as a pragmatic clinical adjunct for baseline phenotyping and longitudinal monitoring in hypothyroid care pathways (see “Clinical implications (selenium)”).

#### Calcium (total/ionized, Ca).

Observation: Ionized Ca contributed to ANN prediction (23.1%) and showed between-group differences in our dataset. Thyroid hormones influence bone turnover and calcium/ phosphate balance; prior work reports negative correlations between TSH and Ca and positive correlations with FT3/FT4 [64]. Our finding that ionized Ca carries predictive information is coherent with these endocrine–mineral interactions and with the cardiometabolic links described for atherogenicity.

#### Magnesium (Mg).

Mg was altered across groups consistent with thyroid dysfunction. Reduced Mg in HT has been frequently reported, with renal Mg loss and immune effects proposed as mechanisms [43], [64]. Given Mg’s vascular and metabolic roles, its changes plausibly track with the IR and dyslipidemic pattern we observed, even when not ranked among the top ANN predictors.

### 5.3 Correlations between depression scores and biomarkers

Another notable outcome of this study is the observed correlations between depression scores and several measured biomarkers. Past research also shows similar associations between depression and thyroid markers. Depression has been found to inversely correlate with FT4, FT3, and T3 across both younger and older adults [74]. Moreover, depression scores significantly relate to levels of TPOAb and TGAb in autoimmune thyroid conditions [75]. HT and depression have been linked in observational studies [76], with evidence indicating gender and age-dependent correlations — particularly in females and younger populations — between TPOAb levels and depressive symptoms [74]. Reduced SEPP levels are associated with more severe depressive symptoms, and selenium supplementation has been shown to lower TPOAb levels [73].

Evidence suggests that even subtle changes in thyroid function within the normal range may elevate depression risk in large cohort studies [77]. While the link between thyroid status and mood is well-established, a deeper understanding of hormone effects on neurotransmitter dynamics is essential. Thyroid hormones regulate the synthesis and function of key mood-related neurotransmitters like serotonin and dopamine. In addition to the established HT-depression comorbidity [78], recent work by Soheili-Nezhad et al. (2023) shows that HT is positively associated with the development of depressive disorders [79]. Depression is commonly found in thyroid patients, and vice versa; many patients diagnosed with depression exhibit abnormal thyroid function [80], [81]. This may be due to the presence of thyroid hormone receptors in brain regions involved in emotion regulation, such as the cerebral cortex, hippocampus, and amygdala [81].

Insulin resistance (IR) and metabolic syndrome contribute to the pathophysiology of atherosclerosis partly by suppressing nitric oxide synthesis [82]. IR leads to hyperglycemia, oxidative stress, and endothelial dysfunction, exacerbating coronary artery disease [83]. It may also underlie the development of unstable angina (UA) [84], as seen in patients with elevated insulin due to pro-inflammatory cytokines like IL-6 [85]. As confirmed in our study and earlier findings [7], HT is associated with elevated serum glucose levels.

Thyroid hormones influence bone resorption, elevating serum calcium and phosphorus while reducing parathyroid hormone secretion [86]. Changes in calcium, phosphate, and magnesium concentrations are emerging cardiovascular risk factors [87]. Zinc supports thyroid hormone synthesis and function, while magnesium regulates membrane permeability and thyroid secretion. These minerals are both regulated by and regulate thyroid hormone metabolism [7], [63]. Turanjanin et al. (2024) reported reduced magnesium levels in HT patients [64]. Total and ionized calcium, along with magnesium, negatively correlate with TSH and positively with FT3 and FT4 levels [64]. Magnesium has protective cardiovascular effects—reducing blood pressure, promoting vasodilation, and opposing atherosclerosis. Therefore, hypomagnesemia is linked to arrhythmia, hypertension, and coronary vasospasm [88], and is also believed to impair immune tolerance [89].

In HT, calcium mobilization from bone is reduced, leading to lower serum calcium. This triggers elevated calcitonin, promoting phosphate reabsorption and increasing calcium loss via urine [90], [91]. HT patients often show decreased total calcium and elevated phosphate [63], along with reduced magnesium [43]. Increased renal magnesium loss has been cited as a cause of hypomagnesemia in HT [64], [92]. Correlation studies that adjust for confounders show strong negative associations between TSH and total/ionized calcium and magnesium, and positive ones with FT3 and FT4 [64]. Similar findings are reported in other works [43], [93]. For example, Sridevi et al. (2016) found a negative correlation between TSH and calcium, though not with magnesium [94], while Un Nisa et al. (2013) reported negative correlations between magnesium and TSH, and positive correlations with FT3 and FT4 [95]. Wang et al. (2018) also found that low magnesium is linked to increased TPOAb, TgAb, Hashimoto’s thyroiditis, and overt hypothyroidism [96]. Elevated TSH has further been associated with increased lipid oxidation and serum lipids [97], although other work suggests positive correlations between magnesium and T3/T4, and inverse correlations with TSH [43].

### 5.4 Serum-based investigations in hypothyroidism

Our study extends serum-based investigations in hypothyroidism by jointly evaluating insulin resistance, dyslipidemia-derived atherogenic indices, and cation/trace-element profiles using an ANN to prioritize predictors of depressive status. This framework builds on the biochemical and metabolic literature already cited in the present article while diverging from pharmacogenomic approaches that personalize antidepressant therapy in major depressive disorder. Whereas pharmacogenomic models primarily inform treatment selection and mitigation of adverse drug reactions after a depression diagnosis, our objective is pre-treatment risk stratification and mechanistic insight within hypothyroid cohorts—highlighting metabolic pathways (e.g., AIP, distance from CRI-I, ionized calcium, HOMA2 indices) that align with the depressive phenotype in HT patients.

### 5.5 Clinical implications (selenium)

In our cohort, selenium emerged as the most reliable biomarker for differentiating hypothyroid patients from healthy controls. Clinically, this supports three pragmatic uses within hypothyroid care pathways: (i) baseline assessment—incorporating selenium alongside thyroid function tests and lipid/atherogenic indices to strengthen initial phenotyping; (ii) longitudinal monitoring—tracking selenium over time to complement conventional follow-up of thyroid status and metabolic indices; and (iii) risk flagging—using abnormal selenium status as an adjunctive signal to prompt closer evaluation of metabolic and neuropsychiatric comorbidities. While our study does not evaluate causality or intervention effects, these findings indicate that selenium can serve as a practical laboratory anchor for patient stratification and follow-up in clinical settings managing hypothyroidism with potential depressive manifestations.

### 5.6 Scientific contribution

The main contributions of this study can be summarized as follows:

• **Integration of Clinical and Computational Approaches:** This work employs a dual methodology, combining biochemical assays with artificial neural network (ANN) analysis, to identify reliable biomarkers for depression in hypothyroid (HT) patients. This hybrid strategy enhances diagnostic accuracy and clinical relevance.
• **Comprehensive Biomarker Evaluation:** A wide spectrum of biochemical parameters — including lipid profiles, thyroid hormones, atherogenic indices, and trace elements — was analyzed to evaluate their roles in HT and its comorbidity with depression.
• **Identification of AIP as a Key Predictor:** The Atherogenic Index of Plasma (AIP) demonstrated 100% predictive accuracy for depression in HT patients, followed by CRI-I (24.7%), ionized calcium (23.1%), HOMA2IR (22.4%), and HOMA2S% (21.8%), highlighting their significance in clinical screening.
• **Differentiation Between HT and Controls via Selenium:** Selenium was identified as the most effective biomarker for distinguishing between HT patients and healthy individuals, underscoring its potential role in thyroid and neuropsychiatric regulation.
• **Novel Insights into Hormonal and Mineral Interactions:** The study provides new evidence on the relationships between thyroid hormones and serum levels of calcium, magnesium, and trace elements, offering insights into the systemic pathophysiology of HT and its neuropsychiatric manifestations.

## 6 Conclusion

### 6.1 Recapitulation

This study comprehensively investigated the biochemical interplay between insulin resistance, lipid metabolism, trace elements, and cationic biomarkers in hypothyroid (HT) patients, with and without comorbid depression. Our findings highlight a distinct profile of metabolic dysregulation in HT patients, particularly those suffering from depressive symptoms. A pronounced state of insulin resistance and dyslipidemia was observed in all HT patients, which intensified in the HT+Dep group. Additionally, significant alterations were noted in serum levels of key cations (calcium, magnesium) and trace elements (copper, zinc, selenium), suggesting a complex biochemical landscape in thyroid dysfunction and its psychiatric comorbidities.

Artificial neural network (ANN) analysis revealed the atherogenic index of plasma (AIP) as the most powerful predictor of depression in hypothyroid individuals, with a predictive importance of 100%. Other important indicators included Castelli’s Risk Index-I (CRI-I, 24.7%), ionized calcium (23.1%), HOMA2IR (22.4%), and HOMA2S% (21.8%). Notably, selenium emerged as the most robust biomarker distinguishing HT patients from healthy controls.

These results underscore the clinical relevance of multi-biomarker profiling in hypothyroidism management, especially in detecting and preventing psychiatric complications such as depression.

### 6.2 Future work

Future research should focus on validating these findings across larger and more diverse cohorts, including patients in different phases of thyroid dysfunction and from various geographic and ethnic backgrounds. Longitudinal studies are recommended to examine the progression of metabolic and neuropsychiatric changes over time in relation to biomarker fluctuations.

Additionally, clinical trials should be designed to assess the therapeutic impact of correcting trace element deficiencies — particularly selenium, calcium, and magnesium — on depressive outcomes in HT patients. From a technological perspective, expanding the ANN framework using explainable AI models could enhance the transparency and clinical utility of predictive diagnostics in endocrine-psychiatric comorbidities.

Further exploration into the integration of AI-driven biomarker screening with electronic health records (EHRs) may also enable real-time risk assessment, paving the way for proactive and personalized interventions in thyroid-related mood disorders.

## CRediT authorship contribution statement

**Sahira Qasim Al-Baldawi:** Writing – original draft, Methodology, Formal analysis, Data curation, Conceptualization. **Hussein Kadhem Al-Hakeim:** Writing – review & editing, Validation, Supervision, Software, Conceptualization. **Habib Hamam:** Writing – review & editing, Visualization, Validation, Project administration, Funding acquisition. **Ikram Khémiri:** Writing – original draft, Validation, Supervision, Project administration, Methodology, Conceptualization.

## Ethical statement:

The study was conducted in accordance with Iraqi and international ethical and privacy laws. Ethical approval for this study was obtained from the Medical Ethics Committee, University of Kufa, Reference #: MEC-96.

## Human and animal rights:

The study was carried out ethically under the World Medical Association Declaration of Helsinki and in compliance with Iraqi, international, and privacy legislation. Additionally, in line with the Declaration of Helsinki, the Belmont Report, the CIOMS Guidelines, and the International Conference on Harmonisation in Good Clinical Practice (ICH-GCP), our IRB adheres to the International Guidelines for the Protection of Human Research Subjects.

## Availability of data and materials:

Some or all of the raw and analyzed data during the current study are available from the corresponding author upon reasonable request.

## Consent for publication:

The authors confirm that human study participants gave informed consent for the publication of their anonymized findings.

## Funding:

This work was supported in part by CRSNG/NSERC under Grant RGPIN-2025-05918.

## Declaration of competing interest

The authors declare the following financial interests/personal relationships which may be considered as potential competing interests: Habib Hamam reports financial support was provided by Natural Sciences and Engineering Research Council of Canada. If there are other authors, they declare that they have no known competing financial interests or personal relationships that could have appeared to influence the work reported in this paper.

## Data Availability

Data will be made available on request.
